# Pomegranate Juice Metabolites, Ellagic Acid and Urolithin A, Synergistically Inhibit Androgen-Independent Prostate Cancer Cell Growth via Distinct Effects on Cell Cycle Control and Apoptosis

**DOI:** 10.1155/2013/247504

**Published:** 2013-04-24

**Authors:** Roberto Vicinanza, Yanjun Zhang, Susanne M. Henning, David Heber

**Affiliations:** UCLA Center for Human Nutrition, David Geffen School of Medicine, University of California, Warren Hall, 900 Veteran Avenue 1-2-213, Los Angeles, CA 90095-1742, USA

## Abstract

Ellagitannins (ETs) from pomegranate juice (PJ) are bioactive polyphenols with chemopreventive potential against prostate cancer (PCa). ETs are not absorbed intact but are partially hydrolyzed in the gut to ellagic acid (EA). Colonic microflora can convert EA to urolithin A (UA), and EA and UA enter the circulation after PJ consumption. Here, we studied the effects of EA and UA on cell proliferation, cell cycle, and apoptosis in DU-145 and PC-3 androgen-independent PCa cells and whether combinations of EA and UA affected cell proliferation. EA demonstrated greater dose-dependent antiproliferative effects in both cell lines compared to UA. EA induced cell cycle arrest in S phase associated with decreased cyclin B1 and cyclin D1 levels. UA induced a G2/M arrest and increased cyclin B1 and cdc2 phosphorylation at tyrosine-15, suggesting inactivation of the cyclin B1/cdc2 kinase complex. EA induced apoptosis in both cell lines, while UA had a less pronounced proapoptotic effect only in DU-145. Cotreatment with low concentrations of EA and UA dramatically decreased cell proliferation, exhibiting synergism in PC-3 cells evaluated by isobolographic analysis and combination index. These data provide information on pomegranate metabolites for the prevention of PCa recurrence, supporting the role of gut flora-derived metabolites for cancer prevention.

## 1. Introduction

Prostate cancer (PCa) is the second most common cancer and the second leading cause of cancer-related death in men, with over 300,000 cases diagnosed annually in the United States [1] with an increasing incidence worldwide due to the growth and aging of the global population [2]. Approximately 30 percent of men treated for PCa with surgery or radiation have evidence of recurrent disease, and in a subset of men, levels of prostate-specific antigen (PSA) continues to rise after treatment [3]. Under these circumstances, rising PSA represents tumor growth, and men with shorter doubling times of PSA value are presumed to have more rapidly growing tumors [4, 5]. A phase II study examining the effects of pomegranate juice (PJ) in men with rising PSA following surgery or radiation for PCa demonstrated that consumption of 8 ounces of PJ significantly increased the PSA doubling time, from 15 to 54 months, suggesting an inhibitory action of PJ metabolites on PCa cell growth [6].

**Figure 1:**
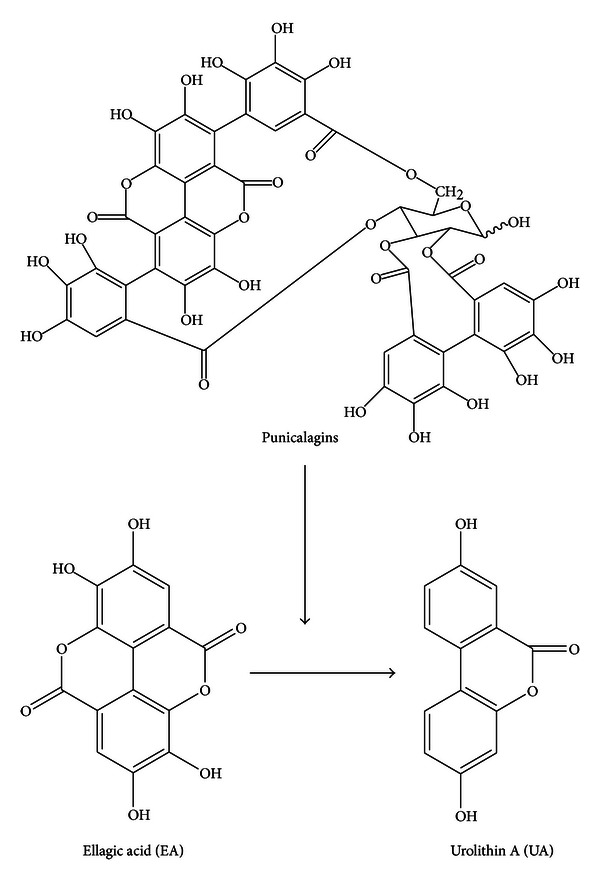
Chemical structures of the major pomegranate ET, punicalagin (occurs as a pair of anomers hence referred to as punicalagins), and its metabolites, EA and UA.

PJ as well as pomegranate extract (PE) contains a family of several high molecular weight (ca.1000 Dalton) hydrolyzable tannins (e.g., punicalagin, punicalin) called ellagitannins (ETs) which have received increasing attention for their potential as nontoxic chemopreventive dietary agents for several malignancies, including PCa [7]. ETs are not absorbed intact in the human gastrointestinal tract, but are hydrolyzed, generating different metabolites including ellagic acid (EA), which appears in the circulation between 30 minutes and 5 hours after consumption of PJ or PE [8, 9]. Through the action of human colonic microflora, EA is partially converted into metabolites including hydroxy-6H-benzopyran-6-one derivatives, primarily urolithin A (UA) (Figure 1). EA and UA are both absorbed, transported in the blood, conjugated in the liver, and excreted in glucuronidated form in the urine between 12 and 56 hours after PJ consumption [10, 11].

Accumulating experimental evidence has demonstrated that PE inhibits tumor angiogenesis [12], delays the transition from androgen-dependent to androgen-independent phenotype, and induces apoptosis through a nuclear factor-kB-dependent mechanism *in vitro *and in tumor tissue excised from castrated severe combined immunodeficiency (SCID) mice with Los Angeles Prostate Cancer-4 (LAPC-4) human PCa xenografts [13]. Previous *in vitro *studies have shown that pomegranate metabolites, EA and UA, inhibited PCa cell proliferation [14, 15] and affected multiple signaling pathways in several cell types [16–18]. For instance, UA decreased both activity and expression of tumor-specific cytochrome P450, CYP1B1, in 22Rv1 PCa cells [19], while EA induced apoptosis via a caspase-dependent pathway [15] and cell cycle arrest in G1 phase involving cyclin-dependent kinase inhibitory protein p21 [20]. Furthermore, EA showed a selective antiproliferative activity for cancer cell lines without affecting the viability of human non neoplastic cells [21, 22]. Other *in vitro *studies demonstrated that specific components of PJ inhibited both the growth and the migration of hormone-dependent and hormone-refractory PCa cell growth and their chemotaxis toward stromal cell-derived factor 1*α* (SDF1*α*) [23].

Cell cycle progression is tightly regulated by different cellular proteins which include the cyclin-dependent kinases (CDKs) and their regulatory cyclin partners [24]. Like other forms of cancer, PCa tumor cells have acquired mutations to genes that directly regulate the cell cycle, resulting in an unrestrained cell proliferation [25–28]. Indeed, loss or attenuation of G2/M checkpoint, controlled by cyclin B1/cdc2 (Cdk1) complex, has been shown to be closely involved in neoplastic transformation [24] and deregulated overexpression of cyclin D1 might be related to the evolution of androgen-independent disease in PCa [29, 30]. Therefore, treatment of tumor cells usually results in the breakdown of the cell cycle machinery leading to the inhibition of cell proliferation and induction of apoptosis. In order to improve our understanding on the mechanisms of action of PJ metabolites in androgen-independent PCa cancer cells, the present *in vitro *study was designed to elucidate the effects of EA and its main microflora-derived metabolite, UA, on cell proliferation, cell cycle distribution, apoptosis, and on the expression of cell cycle-related control proteins. Furthermore, we examined the antiproliferative effect of cotreatment of EA and UA to evaluate their potential synergistic, additive or antagonistic interaction.

## 2. Materials and Methods

### 2.1. Chemicals and Antibodies

Ellagic acid was purchased from Sigma (St. Louis, MO, USA), while urolithin A was synthesized in our laboratory as described previously [9]. Both chemicals were dissolved in DMSO and stored at −20°C. The antibodies against cyclin B1, cyclin D1, p-cdc2 (Tyr.15), and *β*-actin were purchased from Cell Signaling (Beverly, MA, USA). The CellTiter-Glo Luminescent Cell Viability Assay kit was purchased from Promega Corporation (Madison, WI, USA). The Annexin V Cell Apoptosis Detection kit was obtained from BD Pharmingen (San Diego, CA, USA).

### 2.2. Cell Culture

Androgen-independent PCa cell lines, DU-145 and PC-3, were obtained from American Type Culture Collection (ATCC, Manassas, VA, USA). Both cell lines were maintained in RPMI-1640 medium complemented with 10% fetal bovine serum, 2% L-glutamine, and 1% penicillin-streptomycin (Gibco-BRL, Frederick, MD, USA). Cells were used below passage 20, maintained at 37°C in a humidified 5% CO_2_ at 37°C, and subcultured at 1 : 4 by trypsinization for 4 min at 37°C (Gibco-BRL). Before each experiment, cells were switched to complete RPMI phenol red-free medium, and the control groups were treated with appropriate amount of DMSO.

### 2.3. Cell Growth Inhibition Assay and Combination Data Analysis

Cell proliferation was determined using CellTiter-Glo (ATP) assay (Promega) according to the manufacturer's instructions. Briefly, cells were seeded into 96-well opaque-walled plates at a density of 12,000 cells per well in 100 *μ*L of RPMI phenol red-free complete medium. After 24 hours, the medium was carefully removed and the cells were treated in complete medium for 24, 48, 72, 96, and 120 hours with EA (from 15 to 60 *μ*mol/L), UA (from 15 to 90 *μ*mol/L) or DMSO only. The effects of cotreatments of EA and UA were evaluated at 72 hours by treating PC-3 cells with EA (from 1.875 to 30 *μ*mol/L) and UA (from 5 to 180 *μ*mol/L). The tested concentrations were consistent with other *in vitro *studies [14, 15, 21]. The amount of DMSO was normalized in all treatments. At the indicated time point, cells were kept for 30 minutes at room temperature, and 100 *μ*L of the assay reagent was added into each well. The content was mixed for 2 minutes using an orbital shaker to induce cell lysis. After 10 minutes of incubation at room temperature, the luminescence was read using a Microplate Reader (SpectraMax Gemini EM, Molecular Devices, Sunnyvale, CA, USA). The calculations for the IC_40_ and IC_50_ were then performed. Combination effect was evaluated by determining the combination index (CI) using the isobologram equation derived by Chou and Talalay [31]. In general, a CI value <0.9 indicates synergism, a value between 0.9 and 1.1 indicates additivity, and a CI value >1.1 indicates antagonism. In the isobologram, *D*_1_ and *D*_2_ represent the doses of EA and UA alone required to produce the inhibition of cell growth by 40 percent (IC_40_), and *d*_1_ and *d*_2_ are the doses of EA and UA in combination required to produce the same effect (IC_40_). The area on the right side of IC_40_ additive line represents the antagonist effect, while the left side represents the synergic effect.

### 2.4. Protein Extraction and Western Blot Analysis

DU-145 and PC-3 were plated on 60 mm dishes in complete RPMI phenol-red-free medium. Cells were treated with different concentrations of EA (15, 30, and 45 *μ*mol/L) or UA (30, 60, and 90 *μ*mol/L) for 48 and 72 hours. After treatment, cells were washed with cold PBS, harvested, and cell lysates were prepared in RIPA buffer (50 mM Tris HCl pH 7.4, 150 mM NaCl, 2 mM EDTA, 1% Triton, and 0.1% SDS), containing protease inhibitors cocktail (1% v/v) (Sigma-Aldrich), phosphatase inhibitors cocktail (1% v/v) (Sigma-Aldrich), and 20 mM N-ethylmaleimide (Sigma-Aldrich), for 40 min at 4°C. Cell lysates were centrifuged for 10 minutes at 14,000 rpm at 4°C, and the supernatants were collected. Cell lysates were analyzed for protein content by using BCA protein assay with bovine serum albumin as standard. Lysates were then mixed with 4X LDS buffer (Invitrogen) and heated for 10 min at 70°C. 

A total of 55–65 *μ*g of whole-cell protein lysates was separated using 4–12% SDS-PAGE (NUPAGE, Invitrogen), transferred to PVDF membrane, and blocked for 1 hour in 5% milk in T-TBS, containing 0.1% Tween-20 (Sigma). The PVDF membranes were incubated overnight at 4°C with the different primary antibodies in 5% milk in T-TBS and then treated with specific horseradish peroxidase-conjugated anti-rabbit or anti-mouse secondary antibodies. *β*-Actin was used as the internal loading control. Densitometric measurements of the bands in western blot analysis were performed using Quantity One software (Bio-Rad).

### 2.5. Cell Cycle Analysis

DU-145 and PC-3 cells were plated in 100 mm dishes in red phenol-free RPMI complete medium and treated with or without different concentrations of EA or UA for 48, 72, and 96 hours. Following 2 minutes incubation in trypsin, both adherent and floating cells were collected. A number of 1 × 10^6^ cells were then spun down and washed once with cold PBS. Each pellet was resuspended in 500 *μ*L of hypotonic PI staining solution containing 0.1% (w/v) sodium citrate, 0.3% (v/v) Triton X-100, 0.1 mg/mL PI, and 0.02 mg/mL ribonuclease A. Samples were kept in the dark at 4°C for 20 minutes before acquisition. Acquisition was performed on a FACScan flow cytometer with CellQuest software. A total of 20,000 events per sample were acquired. Data analysis was performed using ModFit LT software (Verity Software House, Inc., Topsham, ME, USA).

### 2.6. Detection of Apoptosis

#### 2.6.1. Annexin V/PI Staining

DU-145 and PC-3 cells were plated in 100 mm dishes in red phenol-free RPMI complete medium and then treated with or without the different concentrations of EA (15, 30, and 45 *μ*mol/L) or UA (30, 60, and 90 *μ*mol/L) for 48 and 72 hours. After treatments, both adherents and floating cells were collected. A number of 1 × 10^6^ cells were separated by centrifugation, and the pellet was washed once with cold PBS. Apoptotic cells were identified with Annexin V-FITC and PI double staining, using the Annexin V Cell Apoptosis Detection Kit, BD Pharmingen (San Diego, CA, USA), according to the manufacturer's instructions. After staining, samples were incubated in the dark for 20 minutes at 4°C, and flow cytometric analysis was performed. Data acquisition and analysis were performed with FACScan cytometer (Becton Dickinson, San Jose, CA, USA) using CellQuest software. Cells in early stages of apoptosis were Annexin V positive and PI negative, whereas cells in late stages of apoptosis were both Annexin V and PI positive.

### 2.7. Statistical Analysis

All data are reported as mean ± SD and are representative of at least three independent experiments. Statistical analysis was performed using ANOVA and Tukey's post hoc with Statistica software (Stat Soft Inc., Tulsa OK, USA). The difference between groups was considered statistically significant at *P* < 0.05.

## 3. Results 

### 3.1. EA and UA Differently Inhibit Cell Proliferation of DU-145 and PC-3 Prostate Cancer Cells

**Figure 2:**
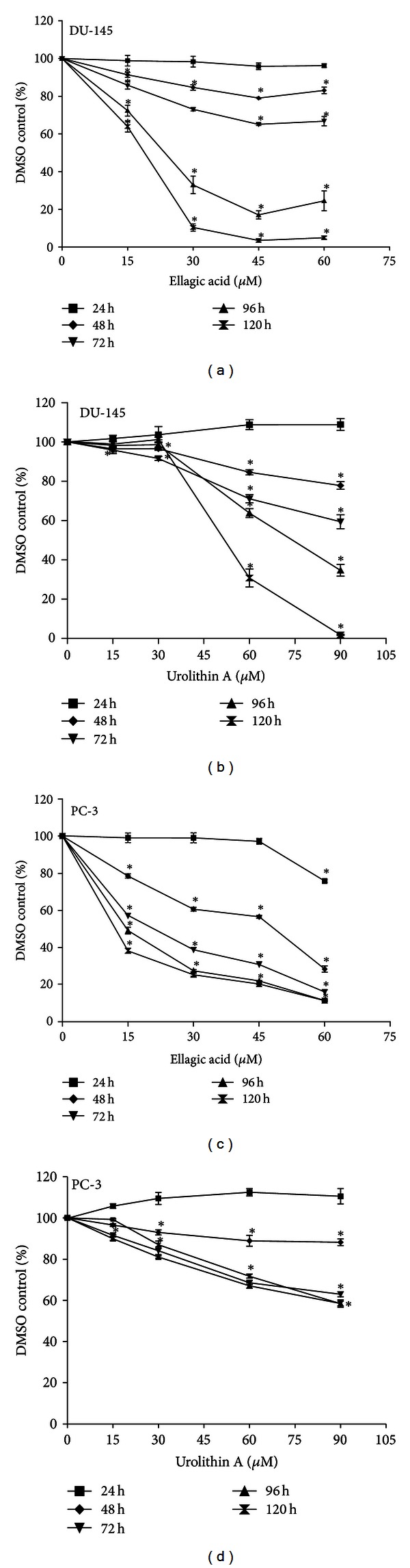
EA and UA inhibited proliferation of DU-145 and PC-3 cells. Cells were seeded into 96-well opaque-walled plates at a density of 12,000 cells. After 24 hours, cells were treated for 24, 48, 72, 96, and 120 with 0, 15, 30, 45, and 60 *μ*mol/L of EA or with 15, 30, 60, and 90 *μ*mol/L of UA. Following treatments with EA, in DU-145 the IC_50_ at 96 hours was 23.02 ± 2.2 *μ*mol/L, while in PC-3 the IC_50_ was 48.33 ± 1.2 *μ*mol/L at 48 hours and 14.5 ± 1.5 *μ*mol/L at 96 hours (a, c). Following treatments with UA, in DU-145 the IC_50_ value at 96 hours was 74.79 ± 2.4 *μ*mol/L (b), while in PC-3, UA did not inhibit the cell growth of 50 percent. Each experiment was performed in triplicate, and the results represented the means ± SD; *significantly different from DMSO control, *P* < 0.05.

The sensitivity of cell growth inhibition in the presence of increasing concentrations of EA (from 15 to 60 *μ*mol/L) and UA (from 15 to 90 *μ*mol/L) was examined at 24, 48, 72, 96, and 120 hours in DU-145 and PC-3, two androgen-independent PCa cell lines. The treatments with EA and UA decreased cell growth in both cell lines in a time-dependent and dose-dependent manner (Figure 2). However, the tested concentrations of EA and UA exhibited differential effects in the two cell lines. Following treatments with EA, the IC_50_ at 96 hours was 23.02 ± 2.2 *μ*mol/L in DU145 cells, while in PC-3 the IC_50_ was obtained at a concentration of 48.33 ± 1.2 *μ*mol/L at 48 hours, and 14.5 ± 1.5 *μ*mol/L at 96 hours (Figures 2(a) and 2(c)). Following treatments with UA, at 96 hours IC_50_ was 74.79 ± 2.4 *μ*mol/L (Figure 2(b)) in DU-145 cells, while in PC-3 UA did not reach 50 percent inhibition of cell growth at the tested concentrations (Figure 2(d)). 

These data indicate that PC-3 cells were more sensitive than DU-145 to EA treatments, while UA treatments exhibited stronger antiproliferative effects in DU-145 compared to PC-3 cells.

### 3.2. EA and UA Induce Cell Cycle Arrest in S and G2/M Phases

**Figure 3:**
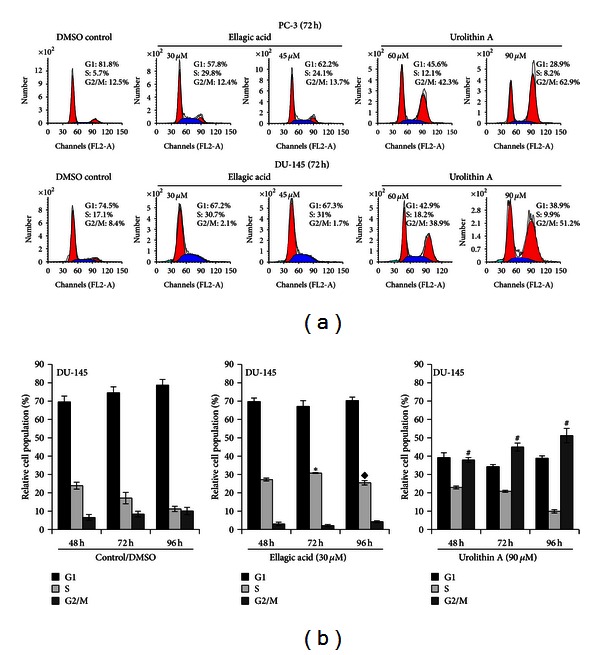
EA and UA induce cell cycle arrest in S and G2/M phases. Representative flow cytometry histograms of cell cycle alterations at 72 h treatments of PC-3 and DU-145 with EA (30 and 45 *μ*mol/L) and UA (60 and 90 *μ*mol/L) (a). Effects of 30 *μ*mol/L EA and 90 *μ*mol/L UA on cell cycle at 48, 72, and 96 h, expressed as the mean of three experiments ± SD of relative cell population (%) (b); *significantly different from S-phase in DMSO/control at 72 h, *P* < 0.01; ^◆^significantly different from S phase in DMSO/control at 96 h, *P* < 0.01; ^#^significantly different from G2/M phase in DMSO/control at 48, 72, and 96 h, *P* < 0.01.

The observed differences of EA and UA treatments on cell proliferation suggested different mechanisms of action of these two compounds. By using FACScan flow cytometer, we first investigated whether EA and UA could affect the cell cycle. Following treatments with 30 and 45 *μ*mol/L of EA for 72 hours, both DU-145 and PC-3 showed a significant increase of cells in the S phase compared to the DMSO control (DMSO control versus EA 30 *μ*mol/L, in DU-145 cells, 17.10 ± 1.8% versus 30.7 ± 0.4%; in PC-3 cells, 5.7 ± 0.7% versus 29.8 ± 1.1%, both *P* < 0.01 versus control). On the other hand, treatment with 60 and 90 *μ*mol/L of UA induced a significant cell accumulation in G2/M phase (UA 90 *μ*mol/L, in DU-145, 51.2 ± 2.8%; in PC-3 cells, 62.9 ± 2.6%, both *P* < 0.01 versus control (Figure 3)). The treatment with EA resulted in a reduction in the percentage of cells in the G1 and G2, while UA resulted in a reduction in the percentage of cells in G1 and S phase. These events were observed at 48, 72, and 96 hours indicating that the effects of EA and UA on cell cycle persisted over 96 hours.

**Figure 4:**
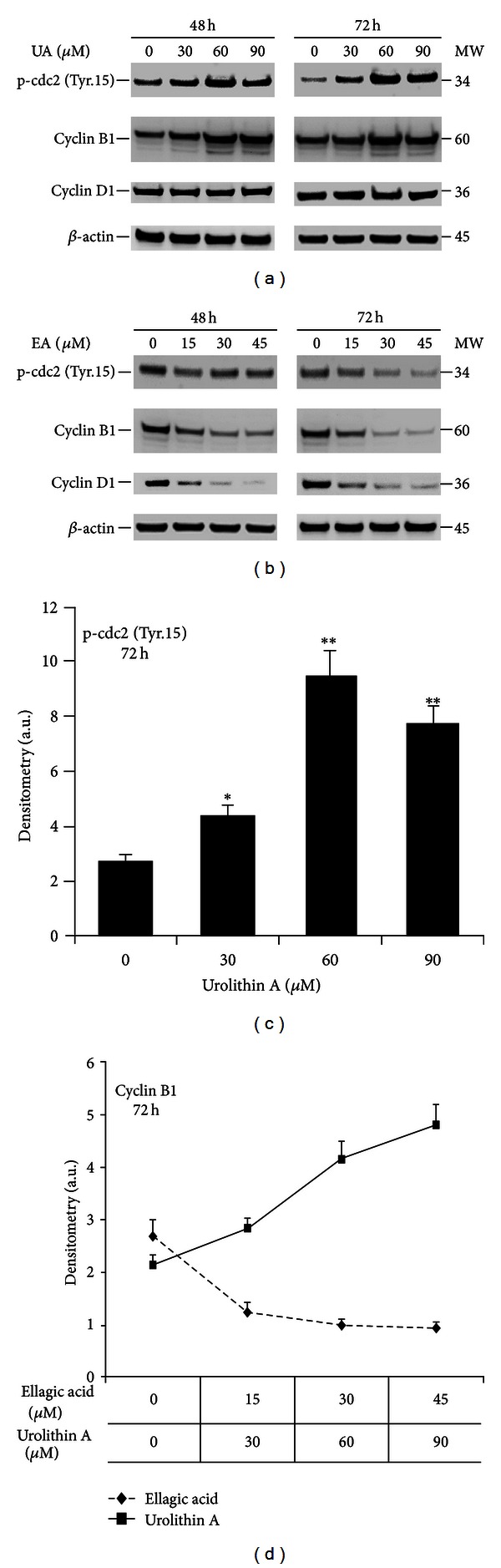
Effect of EA and UA on protein expression of cell cycle regulatory molecules. DU-145 cells were cultured as described Section 2 and treated with 30, 60, and 90 *μ*mol/L of UA or with 0, 15, 30, and 45 *μ*mol/L of EA (b) for 48 and 72 hours. Representative western blots from 3 independent experiments showing the different effects of EA and UA on cyclin B1, Cyclin D1, and phospho-cdc2 at Tyrosin-15 (a, b). Quantification of bands was performed by densitometric analysis (c, d). Data are reported as mean of three independent experiments ± S.D; *significantly different from DMSO/control, *P* < 0.01; **significantly different from DMSO/control, *P* < 0.001.

In order to study the molecular mechanisms involved in the observed effects of EA and UA on cell cycle, the changes in cyclin D1, cyclin B1, and cdc2 phosphorylation at tyrosine-15 were investigated by western blot. The activation of cyclin B1/cdc2 complex (also called mitosis promoting factor (MPF)) is a necessary cell cycle step to promote the entrance into mitosis from the G2 phase, and the cyclin B1/cdc2 complex is controlled by a series of reversible phosphorylations of cdc2 [32]. Phosphorylation of cdc2 at threonine-161 by cdk-activating kinase (CAK) and dephosphorylation of cdc2 at tyrosine-15 by the cdc25C phosphatase are required for the MPF activation [33]. On the other hand, if tyrosine-15 phosphorylation of cdc2 by Wee/Mik1/Myt1 tyrosine kinases occurs, MPF remains inactive leading to G2/M cell cycle arrest [34, 35]. Following treatments for 48 and 72 hours, at concentrations of 30, 60, and 90 *μ*mol/L, UA induced cdc2 phosphorylation at tyrosine-15 and an accumulation of cyclin B1 (Figure 4(a)), consistent with the observed G2/M cell cycle arrest. On the other hand, at concentrations of 15, 30, and 45 *μ*mol/L, EA treatments induced a decrease of cyclin B1 and cyclin D1 expression, consistent with S-phase arrest, with no evidence of tyrosine-15 phosphorylation of cdc2 (Figure 4(b)). Taken together, these data demonstrate that EA and UA have distinct effects on the modulation of cell cycle regulatory proteins leading to arrest the cell cycle in two different phases.

### 3.3. Effect of EA and UA on Apoptosis

**Figure 5:**
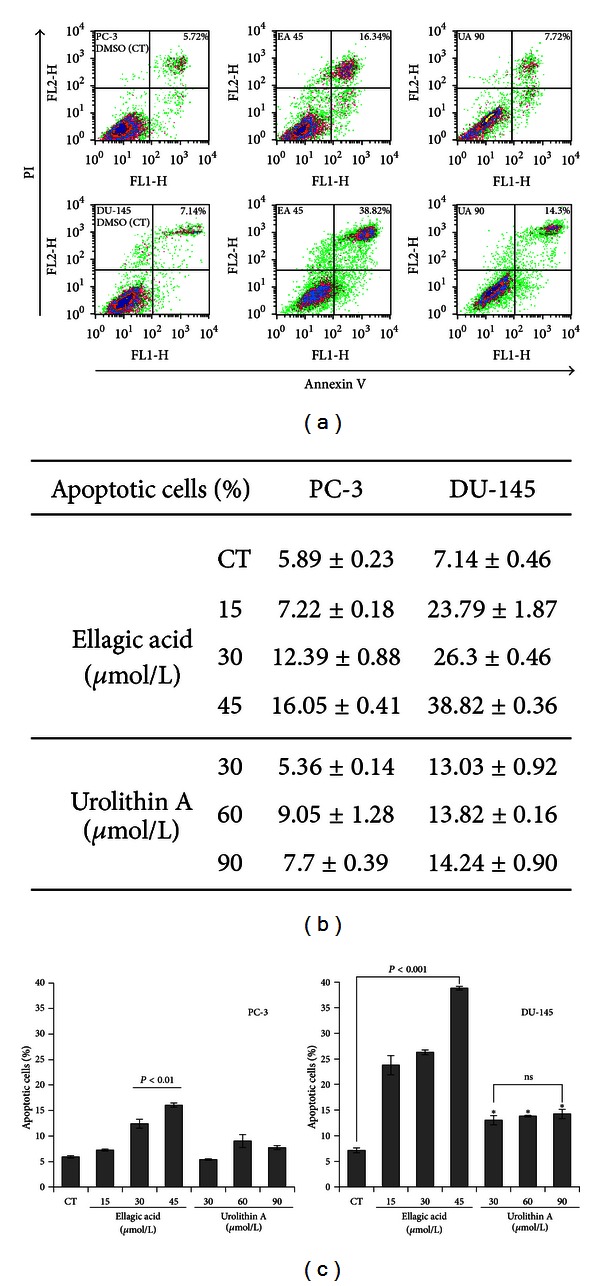
Effect of UA and EA on apoptosis. PC-3 and DU-145 cells were cultured to 85% of confluence in 100 mm dishes and treated with increasing concentrations of EA or with UA for 72 hours. Adherent and floating cells were collected. Cells were double stained with Annexin V/PI and subjected to flow cytometry assay. Representative Annexin V/PI flow cytometry of PC-3 and DU-145 treated with DMSO control, EA 45 *μ*mol/L, and UA 90 *μ*mol/L (a). Percentage of apoptotic cells of PC-3 and DU-145 treated for 72 hours with 15, 30, and 45 *μ*mol/L of EA or with 30, 60, and 90 *μ*mol/L of UA (b, c). The data are mean ± SD of three independent experiments; *significantly different from DMSO control, *P* < 0.01.

We further examined the effect of EA and UA on apoptosis using the Annexin V-FITC/PI double staining method. Following treatments of DU-145 cells with 15, 30, and 45 *μ*mol/L of EA for 72 hours, the percentage of cells in early and late stages of apoptosis increased in a concentration-dependent manner, compared to control (DMSO control versus EA 45 *μ*mol/L, 7.14 ± 0.46% versus 38.82 ± 0.36%, *P* < 0.001), while treatments of DU-145 with 30, 60 and 90 *μ*mol/L of UA resulted in a less pronounced proapoptotic activity compared to EA (Figure 5), with no significant differences among the tested concentrations (DMSO control versus UA 90 *μ*mol/L, 7.14 ± 0.46% versus 14.24 ± 0.9, *P* < 0.01). In PC-3 cells, EA treatment resulted in a significantly increased number of apoptotic cells only at the highest concentration tested (DMSO control versus EA 45 *μ*mol/L, 16.05 versus 5.89 ± 0.23%, *P* < 0.01), while the highest concentration tested of UA did not cause significant apoptosis (DMSO control versus UA 90 *μ*mol/L, 7.72% versus 5.72%, NS).

### 3.4. Cotreatment of EA and UA Synergistically Inhibit PC-3 Cell Proliferation

**Figure 6:**
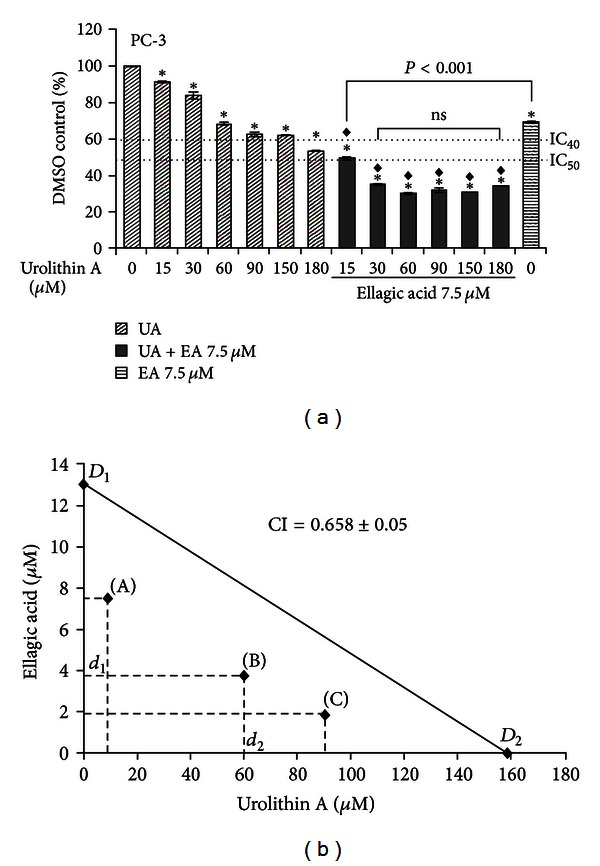
Synergistic effect of EA and UA on proliferation in PC-3 cells. Cells were seeded into 96-well opaque-walled plates at a density of 12,000 cells. After 24 hours, cells were treated for 72 h with 15, 30, 60, 90, 150, and 180 *μ*mol/L of UA or with 7.5 *μ*mol/L alone or in combination (a). The data are mean ± SD of three experiments; *significantly different from DMSO/control, *P* < 0.01; ^◆^significantly different from EA 7.5 *μ*mol/L, *P* < 0.001. Isobologram analysis of the effect of cotreatments of three concentrations of EA and UA, and mean of their combination index (CI) calculating using the classic isobologram equation derived by Chou and Talalay. In the isobologram, *D*_1_ and *D*_2_ represent the doses of EA (*D*_1_) and UA (*D*_2_) alone, required to produce the IC_40_, and *d*_1_ and *d*_2_ are the doses of EA and UA in combination required to produce the same effect (IC_40_). The area on the left side of IC_40_ additive line represents a synergistic effect (b).

Since EA and UA caused a different cell cycle and apoptosis responses, we further investigated the combination of different doses of UA and EA on PC-3 cell proliferation seeking evidence of a synergistic, additive, or antagonistic interaction. We first evaluated the effect of increased concentrations of UA (from 15 to 180 *μ*mol/L) alone or in combination with 7.5 *μ*mol/L of EA at 72 hours. As showen in the Figure 6(a), none of the tested doses of UA induced the IC_50_. However, treatments with low concentrations of EA and UA in combination significantly decreased cell proliferation compared to control, as well as compared to the individual doses of EA and UA (Figure 6(a)). Furthermore, PC-3 cells were treated for 72 hours with different concentrations of EA (from 1.875 to 30 *μ*mol/L) and UA (5 to 180 *μ*mol/L), alone or in combination. Since the maximal inhibition of cell growth in PC-3 cells achieved with UA was 40 percent at a concentration of 158 *μ*mol/L, we used the IC_40_ for the isobolographic analysis of synergy rather than the typical IC_50_. Therefore, the IC_40_ was calculated and the synergistic effect was evaluated using isobolographic analysis. The results of the isobologram equation, using three different concentrations of EA and UA, each dose inhibiting PC-3 cell growth by 40 percent (IC_40_), demonstrated a synergistic inhibitory interaction of EA and UA as shown in the Figure 6(b), with a mean of three CI equal to 0.658 ± 0.05. 

## 4. Discussion

PJ as well as PE contains hydrolyzable ETs which act to inhibit cancer cell growth [36]. These ETs, when ingested, are hydrolyzed rapidly in the intestine and lead to a rise in EA in the blood that begins 30 minutes after consumption and lasts several hours [8, 9]. EA is partially converted in the intestine into other bioactive metabolites, including UA which is metabolized by phase II enzymes and excreted in human urine for up to 48 hours after consumption of PJ [8, 10, 11]. In an animal model, following oral administration of synthesized UA, high levels of UA were detected in mouse prostate tissue [14] suggesting a potential role of UA for prostate health. However, the bioavailability and metabolism of ET metabolites present in pomegranate, as well as in other fruits, are dependent on colonic microflora, and full understanding of these variables is the subject of ongoing investigations. The ET have multiple targets of action in PCa including NF-*κ*B activation, angiogenesis, and androgen synthesis [12, 13, 37]. Therefore, similar to other botanicals, the biological activity does not result from a single ET but is the product of multiple tannins found in the natural product. In turn, these ETs have multiple effects. However, the ingested and absorbed ETs are not the only potential source of biological activity. As with soy isoflavones and green tea, the metabolic products of gut bacterial metabolism also demonstrate antiproliferative activity in cancer cells [38–40].

The possibility of a synergy between ingested PJ phytochemicals and metabolites of the microbiome is a novel concept not previously explored and inspired the present analysis of the antiproliferative actions of EA and its major metabolite UA. 

In this work, we demonstrated that EA and UA both inhibit androgen-independent PCa cell proliferation in a time- and dose-dependent manner. However, the mechanisms of action of UA and EA were characterized by their ability to modulate different intracellular molecular targets, ultimately inducing a distinct effect on cell cycle control and apoptosis. G1/S and G2/M checkpoints are key steps to modulate the passage of cells through the cell cycle and a loss in these checkpoints is critical for the carcinogenesis process [41, 42]. Our experiments showed that UA induced cell cycle arrest in G2/M phase as a predominant mechanism with virtually no effect on apoptosis, while, in addition to cell cycle arrest in S phase, EA also exhibited a significant pro-apoptotic activity in both cell lines with a greater effect in DU-145. In fact, treatments of DU-145 with 45 *μ*mol/L of EA for 72 hours induced 38.82 ± 0.36% of apoptosis (versus control 7.14 ± 0.46%), while in PC-3 the percentage of apoptotic cells was 16.05 ± 0.41% (versus control 5.89 ± 0.23%) as shown by Annexin V/PI double staining. This difference in apoptotic response between these two cell lines likely represents the underlying difference in their molecular components as showen by previous *in vitro* studies demonstrating that DU-145 and PC-3 respond differently to the proapoptotic stimulus, even though both DU-145 and PC-3 are androgen-independent cell lines [43, 44]. Skjøth and Issinger suggested that an impairment of PTEN/AKT pathway, together with low p38MAP kinase, found in PC-3 cells, could be responsible for the observed resistance of PC-3 cells to the induction of apoptosis, whereas a functional PTEN/AKT pathway, as found in DU145, would facilitate the entry of cells into apoptosis [44]. Molecular analysis of selected cell cycle regulatory proteins showed that the effects of UA and EA on G2/M and S phases were associated with a different expression kinetics of cell cycle regulatory molecules such as cyclin D1, cyclin B1, and phosphorylation of cdc2 at tyrosine-15. During the G2/M checkpoint, cdc2 binds to cyclin B1 forming the cyclin B1/cdc2 complex which is the principal enzymatic activity responsible for the initiation of mitosis [45, 46]. However, the complex formation is not sufficient to regulate the initiation of mitosis. Dephosphorylation of cdc2 at the tyrosine-15 site through cdc25c phosphatase is a crucial event for activation of this complex while cdc2 phosphorylation at tyrosine-15 induces its inactivation [47, 48]. In our experiments, we demonstrated that accumulation of cells at G2/M phase by UA treatments for 48 and 72 hours was associated with a persistent increase in cdc2 phosphorylation at tyrosine-15 strongly suggesting the inhibition of cyclin B1/cdc2 complex and therefore the arrest in the G2 phase. This was also confirmed by the presence of cyclin D1 protein expression, which is commonly accumulated in G2 phase, maintained in G1, and suppressed in S phase [49, 50]. Our results are congruent with previous studies demonstrating that other dietary botanical agents, such as apigenin and sulforaphane, inhibit the cyclin B1/cdc2 complex by increasing phosphorylation of cdc2 despite elevated levels of cyclin B1 [49, 50]. On the other hand, consistent with the accumulation of cells in S phase, EA decreased the expression of cyclin D1 and cyclin B1, with no evidence of increased levels of cdc2 phosphorylation at tyrosine-15. Cyclin D1, together with its binding partners CDK4 and CDK6, promote cell cycle progression [51, 52], but recent studies demonstrated that cyclin D1 may also function as transcriptional modulator by regulating the activity of several transcription factors independently of CDK4 activity [53]. The overexpression of Cyclin D1 is a common event in cancer cells and can be caused either by a gene amplification or by a defective regulation at the posttranslational level [54, 55]. 

Therefore, the importance of cyclin D1 in cancer makes it an attractive target for chemoprevention, and several naturally derived compounds may induce cyclin D1 degradation in cancer cells [56]. 

These effects of EA and UA on cell cycle control and apoptosis might therefore explain the different antiproliferative activity of individual doses of EA and UA, as well as the synergistic effect of low concentrations of EA and UA in combination in PC-3 cells. 

In fact, as shown in Figure 6(a), 7.5 *μ*mol/L of EA combined with 15 *μ*mol/L of UA finally induced cell growth inhibition of 50 percent, while none of the tested doses of UA induced the IC_50_. The potential synergistic effect between EA and UA was further investigated and confirmed by the isobolographic analysis and by the calculation of the CI which was less than 0.9 (0.658 ± 0.05) as shown in Figure 6(b). 

## 5. Conclusions

In a phase II clinical trial, PJ led to a prolongation of doubling time of rising serum PSA in men with recurrent PCa, consistent with a direct effect of PJ metabolites on PCa cell growth [3]. 

The interpretation of increased serum PSA levels is complicated by potential origin of PSA from both normal cells and PCa cells, while a rising PSA level confirmed over several months in men with recurrent PCa derives exclusively from PC cells, and PSA levels are used as an indicator of disease progression [57]. The complex nature of human PCa and the lack of clear biomarkers make primary prevention studies of nutrition interventions extremely difficult. Therefore, the challenge in the prevention of PCa recurrence is the inhibition of androgen-independent tumor growth after primary treatment. However, because of the diversity of advanced PCa and its capacity to adapt to changing conditions, targeting a single pathway may not ensure long-term effects since PCa cells may activate surrogate kinases or alternative pathways. In turn, cancer cells may develop resistance to targeted inhibitors. Consequently, inhibition of multiple pathways may be an encouraging strategy to avoid adverse effects connected with target redundancy.

In the present study, we report that PJ metabolites inhibit cell proliferation of PCa cells through two distinct mechanisms of action, and they may interact synergistically to inhibit androgen-independent PCa cell growth. This synergism suggests that the potential chemopreventive source of PJ may also be deriveed from the products of gut flora metabolism which can further amplify the antiproliferative effects of specific bioactive components contained in PJ. Furthermore, our results provide additional insight into the mechanisms of action of PJ metabolites for the prevention of PCa recurrence.

## Abbreviations

EA: Ellagic Acid
ET: Ellagitannins
PJ: Pomegranate juice
PE: Pomegranate Extract
PCa: Prostate Cancer
UA: Urolithin A.
